# Mitochondrial Dynamics and the ER: The Plant Perspective

**DOI:** 10.3389/fcell.2015.00078

**Published:** 2015-12-23

**Authors:** Stefanie J. Mueller, Ralf Reski

**Affiliations:** ^1^Plant Biotechnology, Faculty of Biology, University of FreiburgFreiburg, Germany; ^2^BIOSS Centre for Biological Signalling Studies, University of FreiburgFreiburg, Germany; ^3^FRIAS Freiburg Institute for Advanced Studies, University of FreiburgFreiburg, Germany; ^4^USIAS University of Strasbourg Institute for Advanced Study, University of StrasbourgStrasbourg, France

**Keywords:** organelle dynamics, *Physcomitrella patens*, fission, fusion, *Arabidopsis thaliana*

## Abstract

Whereas contact sites between mitochondria and the ER have been in the focus of animal and fungal research for several years, the importance of this organellar interface and the molecular effectors are largely unknown for plants. This work gives an introduction into known evolutionary differences of molecular effectors of mitochondrial dynamics and interactions between animals, fungi, and plants. Using the model plant *Physcomitrella patens*, we provide microscopic evidence for the existence of mitochondria-ER interactions in plants and their correlation with mitochondrial constriction and fission. We further investigate a previously identified protein of unknown function (MELL1), and show that it modulates the amount of mitochondrial association to the ER, as well as mitochondrial shape and number.

## Introduction

Subcellular compartmentation has enabled eukaryotes to simultaneously establish distinct reaction compartments with discrete protein content that need to be coordinated by interorganellar communication. Compartments are linked by signaling pathways and transport processes of different types of molecules such as proteins, lipids, and carbohydrates. Increasing evidence suggests that these processes are coordinated at specific contact interfaces (Prinz, 2014) which are either modulated by proteins or even membrane hemifusions (Mehrshahi et al., 2013, 2014). Multiple effectors of membrane contact sites (MCS) were identified linking the omnipresent ER to most other cell compartments, as e.g., the plasma membrane, lysosomes, vacuoles, and to mitochondria in mammals and yeast (for review, see Prinz, 2014). Identified functions of MCS include the transfer of lipids and the regulation of intracellular Ca^2+^ in animals and fungi (Prinz, 2014), and the accessibility to nonpolar metabolites between plant ER and plastids (Mehrshahi et al., 2014). In particular, the interactions between mitochondria and ER became a focus of research during the last decade in animals and fungi, linking ER-mitochondria contacts additionally to mitochondrial dynamics and quality control (Rowland and Voeltz, 2012; Kornmann, 2013; Lackner, 2014).

Mitochondria of a single cell have been described as a discontinuous whole (Logan, 2006), as they undergo frequent fusion and fission in animals, fungi, and plants (Arimura et al., 2004; Labbé et al., 2014), and thus maintain a certain rate of content exchange. This process was recently shown to be important for fatty acid metabolism in mammalian cells under starvation (Rambold et al., 2015), but is best known for its pivotal role in mitochondrial quality control (Twig et al., 2008b). Notably, mitochondrial fusion can either be transient (“kiss-and-run”) while retaining mitochondrial identities, or of longer duration with increased exchange of matrix and also membrane content (Liu et al., 2009).

A model for the mixing and unmixing of mitochondrial content was proposed, describing a separation of dysfunctional mitochondria from the pool of fusing mitochondria, and their targeting to autophagosomes (Twig et al., 2008b). In mammalian cells mitochondrial fusion triggers fission which in turn is followed by selective fusion (Twig et al., 2008b): differences in membrane potential become evident in daughter mitochondria after a fission event (Twig et al., 2008a). As membrane potential and import capacity are linked, the PINK/Parkin pathway subsequently regulates the exclusion of dysfunctional mitochondria via degradation of components of the fusion machinery in mammals (Narendra et al., 2012). A loss of this quality control system can in turn disturb stem cell fate in mammals (Katajisto et al., 2015) and leads to the decrease or the total loss of mitochondrial genomes in yeast and mammals (Labbé et al., 2014).

Although it was known for some time that mitochondrial form and function are linked, i.e., that changes in mitochondrial morphology and/or dynamics often are the first marker for cell stress in mammals, fungi, and plants (Scott and Logan, 2008; Welchen et al., 2014), the identity of several molecular effectors was only discovered in recent years. Thus, several components of the fission machinery are evolutionary conserved, such as dynamin-related GTPases (yeast Dnm1p, mammals Drp1, and *A. thaliana* DRP3A/DRP3B) and FIS-type proteins (*FISSION*, also called *BIGYIN* in plants; Scott and Logan, 2011). Notably, in both yeast and mammalian cells ER-mitochondrial contacts contribute to mitochondrial fission, supposedly either by the physical constriction of mitochondria by ER tubules, or as platforms for recruitment of the fission machinery (Friedman et al., 2011). In yeast, ER-mitochondrial interactions are mediated by the ERMES [ER-Mitochondrial Encounter Structure (Kornmann, 2013)] complex which has no known homologs in mammals or plants (Duncan et al., 2013; Kornmann, 2013).

The fusion machinery of mitochondria is largely conserved between mammals and yeast and involves the dynamin-related GTPases homologous to the FUZZY ONIONS (Fzo) protein from *Drosophila melanogaster*: Fzo1p in yeast and mitofusins (Mfn1, Mfn2) in mammals. These GTPases contain two C-terminal transmembrane domains and mediate tethering of neighboring organelles and outer membrane fusion (Labbé et al., 2014). In mammalian cells Mfn2/Mfn1 interactions additionally regulate mitochondrial/ER tethering and Ca^2+^ uptake (de Brito and Scorrano, 2008). In contrast, in land plants the closest homologs of this protein family localize to chloroplasts and mediate thylakoid architecture (Gao et al., 2006), raising the question how plant mitochondria fuse (Arimura et al., 2004; Scott and Logan, 2011).

Although evidence for links between plant mitochondrial form, function, and dynamics exist, the molecular identity of interaction sites is mostly unclear and modulators and effectors known from animal systems, such as Bcl2-like proteins, mitofusins, PINK, and Parkin (Logan, 2006, 2010; Elgass et al., 2013; Labbé et al., 2014) are lacking. Interestingly, plants possess a highly organized ER with different subdomains including potential contact sites to mitochondria, with suggested functional links to the transfer of lipids and mitochondrial dynamics (Staehelin, 1997; Sparkes et al., 2009; Stefano et al., 2014a).

We recently identified a plant protein with LEA (Late Embryogenesis Abundant) and LysM domains and a conspicuous subcellular localization to ER and mitochondria (MELL1), which influences mitochondrial shape (Mueller et al., 2014). Here, we describe its influence on the association between mitochondria and ER and discuss future challenges in mitochondrial dynamics research.

## Results

**Figure 1:**
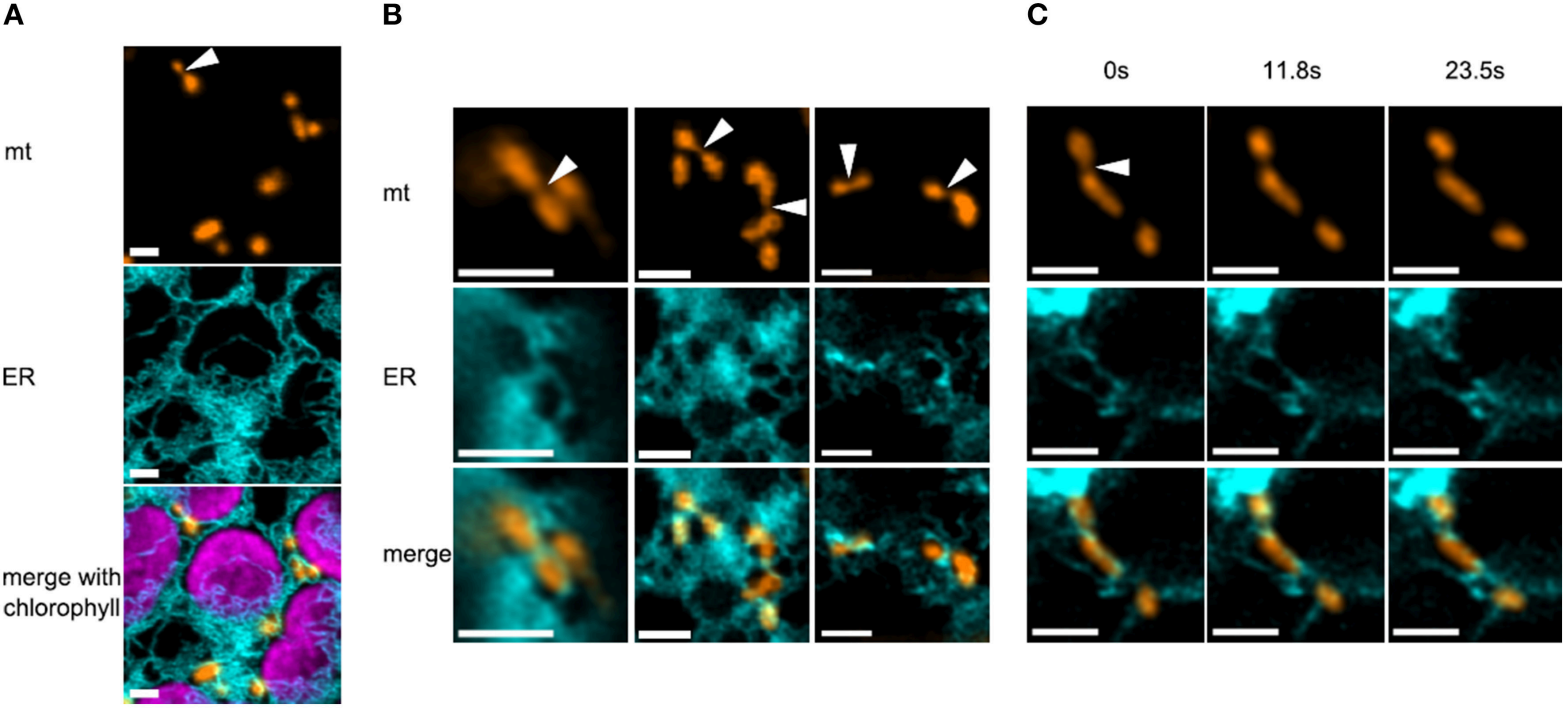
**Correlation between constriction sites in mitochondria and ER tubules in moss. (A)** Overview of several mitochondria, the ER and chloroplasts in a protoplast of *Physcomitrella patens*. ER tubules are often closely associated to both organelles. The arrowhead points to a mitochondrion with a prominent constriction site, co-localizing with ER. Scale bars are 2 μm. **(B)** Several mitochondria show co-localization with the ER at constriction sites (arrowheads) and at their ends. Scale bars are 2 μm. **(C)** Time series (~2 min) of a mitochondrial fission event in moss, showing the close association of ER, which subsequently remains attached on both newly generated ends of daughter mitochondria. Scale bars are 2 μm.

In order to monitor mitochondria and ER simultaneously in a plant, we used fluorescently labeled organelles of the model moss *Physcomitrella patens*, which provides a uniquely high rate of homologous recombination in plants (Strepp et al., 1998) and is amenable to confocal microscopy studies (Abel et al., 1989; Furt et al., 2012; Vidali and Bezanilla, 2012; Müller et al., 2015). We generated a stable transgenic moss line constitutively expressing mitochondria-targeted mEOS (mtEOS; Mathur et al., 2010) and transiently transfected protoplasts of this line with an ER marker that comprises a signal peptide, mCerulean, and a C-terminal KDEL ER retention signal (spCerKDEL). We found that mitochondria in moss protoplasts were mostly small elongated tubular structures which move only at about a 10th of the speed of flowering plant mitochondria (max. speed in our hands was 75 nm/s), which supports previous findings (Pressel et al., 2008; Furt et al., 2012). ER tubules tightly wrapped most mitochondria of a cell (Figure 1A). When investigating high quality images of mitochondria and ER (*n* = 51), a third of the mitochondria showed an elongated shape with clear constriction sites (Figure 1B). In our dataset, 88% of these constriction sites showed a clear co-localization with ER tubules. Mitochondrial constriction sites did not always lead to fission events in the time frame of several minutes. But when fission events occurred (Figure 1C), ER was closely associated and ER tubules remained attached on both newly generated ends of daughter mitochondria. Thus, ER and mitochondrial dynamics are linked in moss, although the causality of this correlation is as yet unclear.

**Figure 2:**
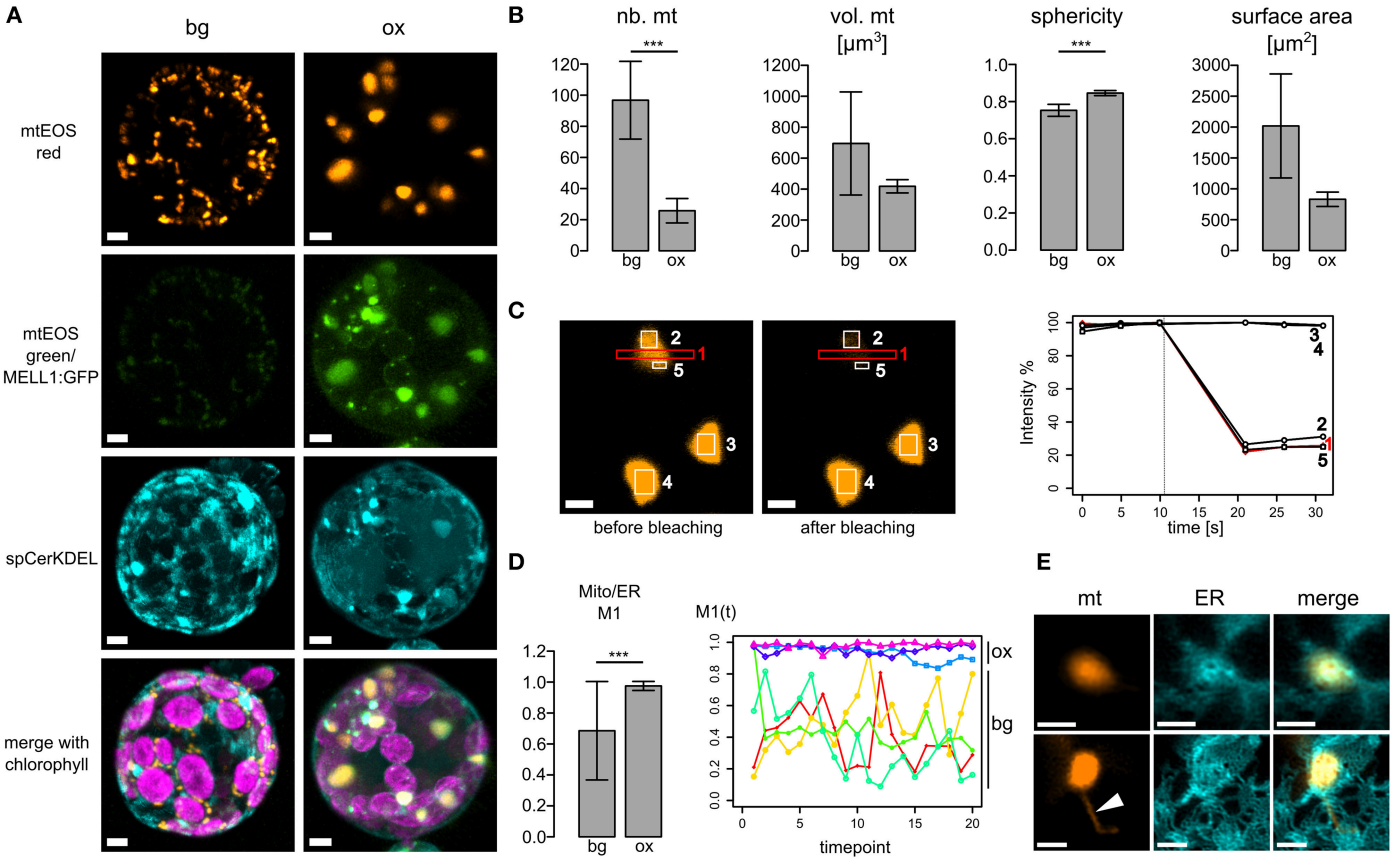
**Overexpression of MELL1 affects mitochondrial shape and association with the ER. (A)** 3D Reconstructions of z-stacks of confocal planes from transfected moss protoplasts. Left panel: protoplast of a stably transformed mtEOS line (background bg) showing normal size and distribution of mitochondria and ER (spCeruleanKDEL). mtEOS is almost completely photoconverted to its red form. Right panel: MELL1:GFP overexpression (ox) leads to a profound change in mitochondrial shape and number, as well as to the accumulation of ER around mitochondria. Scale bars are 4 μm. **(B)** MELL1-overexpressing cells (ox) have significantly less mitochondria (*p* < 0.01, *n* = 4), whereas total mitochondrial volume was not significantly reduced (*p* = 0.23, *n* = 3). Mitochondria show a more spherical shape (*p* < 0.01, *n* = 3) and a concomitant trend to decreased surface area (*p* = 0.07, *n* = 3). **(C)** Large spherical mitochondria in MELL1-overexpressing cells are fused, as shown by photobleaching of mtEOSred (region 1) and a parallel drop in fluorescence intensity in neighboring regions of the same mitochondrion (regions 2+5), but not in other neighboring mitochondria (regions 3+4). **(D)** Mander's coefficient for co-localization of mitochondrial signal (mtEOSred) with ER signal (spCeruleanKDEL). Mitochondria co-localize significantly more with ER in different cells (^***^p < 0.01, *n* = 13 ox, *n* = 6 bg), as well as during time series of the same cell [M1(t), *p* < 0.01, *n* = 3 ox, *n* = 4 bg]. **(E)** Detail of large spherical mitochondria in MELL1-overexpressing protoplast, showing close association with a network of many ER tubules. Arrowhead points to matrixule. Scale bars are 2 μm.

As mitochondria and ER dynamics correlate, we further investigated whether ER-mitochondria association is altered by overexpression of the ER-mitochondria localized protein we recently identified (MELL1; Mueller et al., 2014). Figure 2A depicts 3D reconstructions of a typical protoplast expressing spCerKDEL in the mtmEOS background line (bg) or spCerKDEL and MELL1:GFP in the mtmEOS background line (ox). Mitochondrial shape is severely altered toward large mitochondria. Mitochondrial number is significantly reduced and sphericity of mitochondria significantly increased (Figure 2B), whereas the total volume of mitochondria was not significantly altered (Figure 2B).The increase in sphericity induces a trend toward decreased surface area of the mitochondria, which was not statistically significant in our dataset (Figure 2B).

Large mitochondria possess a common matrix space, as photobleaching of mtEOS lead to a concomitant decrease of fluorescence intensity in neighboring areas of the same mitochondrion (Figure 2C). We tracked the association of mitochondria and ER by Mander's co-localization coefficient (M1 Figure 2D) between different transfected cells (left), and over several time series (right, ~duration 2 min). The co-localization of mitochondria with the ER was significantly increased for cells over-expressing MELL1, compared to cells of the background line. Moreover, the co-localization coefficient remained elevated during the time courses in MELL1 over-expressing cells, in contrast to a higher variance of mitochondria-ER co-localization in the background line. Thus, the association of mitochondria and ER is increased in MELL1 over-expressing cells and shows a high temporal persistency. Figure 2E depicts details of the association between mitochondria and ER under MELL1 overexpression. Mitochondria are embedded in a dense network of ER tubules and occasionally (Figure 2E arrowhead) show tubular extensions [matrixules (Logan, 2006)].

## Discussion

### Connectivity between organelles in plant cells

In plants, the existence of specialized contact domains between the ER and other organelles such as chloroplasts was evidenced by several experimental approaches, either exerting mechanical forces by optical tweezers (Andersson et al., 2007), or using transorganellar complementation to demonstrate biochemical continuity (Mehrshahi et al., 2013). Mitochondria and ER cooperate in several biosynthetic pathways and exchange phospholipids in plants (for review see Millar et al., 2008). However, the molecular identity of proteins mediating contact sites and connectivity between mitochondria and ER in plants is so far unknown.

Studies investigating organelle movement in plants point to the presence of tethers or hemifused membranes between the ER and other organelles, as organelle dynamics correlate, without evidence for luminal connectivity (Stefano et al., 2014a,b). Two factors modulating these interactions may be membrane curvature and shape, as well as movement on cytoskeletal elements (Stefano et al., 2014a). Thus, inhibition of both actin filaments and microtubules was found to promote mitochondrial fusion in plants (Sheahan et al., 2005), probably indicating that movement on cytoskeletal elements counteracts complete fusion, similar to the situation in the mammalian system (Liu et al., 2009). Further, when the actin and the microtubule cytoskeleton were perturbed simultaneously, mitochondria tended to cluster around chloroplasts and ER patches (Van Gestel et al., 2002), implying mechanisms for specific association that lead to typical plant subcellular positioning (Welchen et al., 2014). As we have shown here, mitochondria co-localize with ER in moss protoplasts (Mander's coefficient 0.69 ± 0.32), often with one or several ER tubules crossing parts of the mitochondrial surface and the ends of elongated mitochondria. This co-localization showed a high variance in the mtEOS-labeled line, indicating frequent changes in the amount of ER in the ultimate proximity of mitochondria. Similar to animal and fungal model systems, we found that ER labeled mitochondria constriction sites, suggesting an evolutionary conservation of mitochondria-ER interactions at constriction sites. In order to investigate the molecular basis and effect of this correlation, contact sites between ER and mitochondria in plants await identification, as no homologs to ERMES or mitofusins are present in plant mitochondria.

### MELL1 level influences the association of mitochondria to the ER

In differentiated plant cells, mitochondria undergo frequent fusion and fission (Arimura et al., 2004) without global changes in number or shape, whereas differentiating protoplasts show massive mitochondrial fusions (Sheahan et al., 2005), putatively to redistribute mtDNA. Overexpression of MELL1 led to large fused mitochondria, which were closely associated to a constitutively high amount of ER. In theory, this phenotype could either relate to increased fusion of mitochondria, or decreased fission. Interestingly, though major changes in mitochondrial shape and distribution occurred, mitochondria were not dysfunctional, as indicated by correct targeting of the mtEOS probe. Additionally, the ability to form tubular extensions (matrixules) was retained under MELL1 overexpression.

Using forward and reverse genetics, conserved molecular mechanisms behind mitochondrial fission as well as plant-specific modulators were characterized, such as *NETWORK/ELM* (ELongated Mitochondria) which is required for the localization of DRP3A to plant mitochondria (Arimura et al., 2008). In the model flowering plant *Arabidopsis thaliana*, the evolutionary conserved dynamin-related GTPases DRP3A and DRP3B mediate mitochondrial (and peroxisomal) fission (Fujimoto et al., 2009). Mutations of components of the fission machinery (DRP3A, DRP3B, FIS1A, FIS1B) lead to defects in mitochondrial shape and distribution, resulting in a reduced number of mitochondria with a more spherical shape (Scott et al., 2006; Zhang and Hu, 2008; Fujimoto et al., 2009), similar to our results. Other plant mutants exhibiting an aggregation of mitochondria include FRIENDLY, a homolog to mammalian CLUH (clueless homolog; Gao et al., 2014), which causes clustering of mitochondria and an increase in matrix exchange, but no hyperfusion (El Zawily et al., 2014). CLUH was recently shown to bind mRNA of mitochondrially targeted proteins and may thus influence mitochondrial distribution indirectly via mitochondrial biogenesis (Gao et al., 2014). In MELL1 overexpressing protoplasts, mitochondria underwent complete fusion to large spherical mitochondria, with a common matrix space (Figure 2C) indicating a disturbed balance between fusion and fission. As this effect is accompanied by an increase in the association of ER to mitochondria, MELL1 might either directly or indirectly influence proteins at mitochondria-ER contact sites in plants. Whether MELL1 overexpression causes increased mitochondrial fusion or decreased mitochondrial fission is unclear so far. It is tempting to speculate that the increased mitochondrial association to the ER would disturb the fission machinery, as ER-mediated positional clues for fission, either provided by constriction via ER-tubules, or recruitment of the fission machinery to contact sites (Friedman et al., 2011), might be lacking. Alternatively, MELL1 might be a first link to the unknown mitochondrial fusion machinery in plants, although the protein does not contain a GTPase domain itself. An intriguing possibility is that MELL1 influences membrane curvature, as LEA domains may form alpha-helical structure which insert laterally into membranes (Tolleter et al., 2010; Candat et al., 2014). Future studies of knock-out mutants and mitochondrial dynamics in plants, as well as interacting proteins will address these open questions.

In conclusion, surprisingly little is known about the molecular identity of organelle contact sites in plants, but the evidence presented in this work points to an evolutionary conserved importance of mitochondrial dynamics and contacts to the ER between fungi, animals, and plants, while evolution may have shaped analogous molecular effectors. It will further be interesting to investigate whether there is any common mechanism in mitochondrial fusion shared by all eukaryotes. Future challenges include the identification of candidate proteins for organellar contact sites in plants, to further link changes in organellar form and function to the context of organelle connectivity, and to unravel the mechanisms behind balanced fusion/fission processes and quality control in mitochondria.

## Material and methods

### Cloning

Mitochondria-targeted mEOS (Wiedenmann et al., 2004), containing the first 261 bp of the *Nicotiana plumbaginifolia* mitochondrial ATP2-1 coding sequence (X02868) as N-terminal targeting signal (Logan and Leaver, 2000; Mathur et al., 2010), was amplified via PCR (F ATAAGTCGACATGGCTTCTCGGAGGCTTCT, R ATCCGAGCTCTTATCG TCTGGCATTG) and ligated via the introduced SalI and SacI restriction sites into a newly assembled vector backbone containing the moss *Actin5* promoter (Weise et al., 2006) and a *NOS* terminator, as well as homologous regions for gene targeting to the “*P. patens* targeting site 2” (PTA2; Kubo et al., 2013) locus (*pAct5_PTA2*). To assemble this vector, PTA2 5′ homologous region (F GCTCTTCTCCTGGGGATTAATTATTGGAGG, R GAAA GAACGAATTCGATCGGATCCGCGACTAGTGAGAGAATGTT) and PTA2 3′ homologous region (F CTAGTCGCGGATCCGATCGAATTCGTTCTTTCTGTCATTAAC TGG, R GCTCTTCATTGTTCAGGATAATGGTTC) were amplified from genomic DNA, joined with two template PCR (Tian et al., 2004) and ligated into a pJET1.2 vector (Thermo Fisher Scientific). The expression cassette of *Actin5* promoter, multiple cloning site, fluorescent protein, and *NOS* terminator (Mueller et al., 2014) was subsequently introduced between the PTA2 homologous regions with the restriction enzymes BamHI and EcoRI. To create an ER marker construct, the mCerulean coding sequence was amplified from *pGEMHE-X-Cerulean* (BIOSS toolbox Freiburg), and codons for the ER retention signal KDEL added to the C-terminus (F TACTGTCGACGTGAGCAAGGGC GAGGAG, R TTACAGCTCATCCTTCTTGTACAGCTCGTCCATGC). This construct was introduced in the *pAct5_PTA2* via restriction and ligation using SalI and Ecl136II. Subsequently, the signal peptide from moss aspartic protease (Schaaf et al., 2004) was PCR amplified from genomic DNA (F ATCAGTCGACATGGG GGCATCGAGGAGTGTT; R ATTAGTCGACGCGAGGGCTTGCCTCAGC TA) and introduced in front of the *mCerulean::KDEL* with SalI restriction and ligation.

### Moss protoplast transfection

Moss protoplasts of the *P. patens* (Hedw.) Bruch & Schimp. Gransden strain (International Moss Stock Center IMSC #40001) were prepared and transfected as described previously (Strepp et al., 1998; Hohe et al., 2004; Mueller et al., 2014). For stable transformation, an uncut plasmid containing the *nptII* neomycin resistance cassette (*pBSNNNEV*) was co-transfected in a ratio of 3:1 with the construct for homologous recombination. For transient transfection, *pAct5_PTA2* vectors containing organelle marker constructs and MELL1 overexpression vector (Mueller et al., 2014) were used uncut (10 μg per construct), whereas the construct was released from the vector creating homologous ends via BspQI restriction sites for stable transformation (30 μg used per transfection). Moss protoplasts were kept in the dark and imaged between 48 and 72 h after transfection. A stable mtEOS line (mtmEOS#44) is available from the International Moss Stock Center (IMSC #40776).

### Confocal microcopy and image analysis

All confocal images were taken with a Zeiss LSM 510 META with upright microscope Axio Imager Z1, using a C-Apochromat 63x/1.2 W Korr objective with water immersion. Fluorophores were excited with either an Argon laser (3% 488 nm for GFP/mtEOSgreen/chlorophyll), or diode lasers (3% 561 nm for mtEOSred; 3% 405 nm for Cerulean) using three separate tracks. Fluorescence was detected for chlorophyll from 670–756 nm (false colored magenta), for GFP from 505–550 nm (false colored green), for mtEOSred from 575–615 nm (false colored orange). Pinhole was set to 1 AU for Cerulean channel and section thickness adjusted accordingly in all other channels. Pixel dwell was 1.61 μs. Images were taken using 4 averages and 256 × 256 pixel for time series (~7 s per time point) and using 16 averages and 512 × 512 pixel for snaps. The zoom factor was adjusted to guarantee 1.5-2x overimaging of pixels, as recommended for deconvolution (see Huygens software manual). Bleaching (Figure 2C) settings for mtEOSred were used as follows: start after three scans, 300 iterations of bleaching, 100% 561 nm laser.

Confocal images were all deconvolved prior to subsequent analyses using Huygens Remote Manager (v3.2.2, Scientific Volume Imaging; SNR = 8 for time series, SNR = 10–15 for snaps). Co-localization analysis was performed in Huygens using Mander's coefficient (Manders et al., 1993). Three-dimensional reconstructions of z-stacks were performed after deconvolution using the Imaris software (Bitplane). Mitochondrial number was analyzed using the icy (http://icy.bioimageanalysis.org; de Chaumont et al., 2012) spot detector tool. Volume, shape, and surface area was analyzed by creating a surface from the mtEOSred channel in Imaris (Bitplane) and the surface statistics tool. Statistical analyses were conducted using the GraphPad Software Quickcalcs tools (http://www.graphpad.com/quickcalcs/), using two-tailed *T*-test. Bar graphs show mean and standard deviation.

## Author contributions

SM and RR planned and designed the research. SM performed experiments and analyzed data. SM and RR wrote the manuscript. All authors discussed data and approved the final version of the manuscript.

### Conflict of interest statement

The authors declare that the research was conducted in the absence of any commercial or financial relationships that could be construed as a potential conflict of interest.
